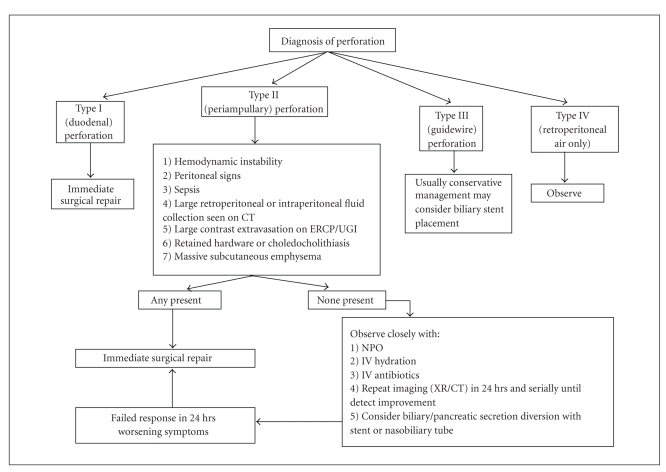# Successful Nonsurgical Treatment of Pneumomediastinum, Pneumothorax, Pneumoperitoneum, Pneumoretroperitoneum, and Subcutaneous Emphysema following ERCP

**DOI:** 10.1155/2010/289135

**Published:** 2010-06-14

**Authors:** L. Fujii, A. Lau, D. E. Fleischer, M. E. Harrison

**Affiliations:** ^1^Mayo Clinic, 13400 E Shea Blvd, Department of Internal Medicine, Phoenix, AZ 85259, USA; ^2^Mayo Clinic, 13400 E Shea Blvd, Division of Gastroenterology and Hepatology, Phoenix, AZ 85259, USA

## Abstract

Complications related to endoscopic retrograde cholangiopancreatography (ERCP) include pancreatitis, hemorrhage, cholangitis, and perforation. ERCP-related perforation is uncommon, but mortality rates are high. Diagnosis requires a high clinical suspicion for early detection to allow optimal management of the perforation and a better prognosis. Treatment depends on the location and mechanism and increasingly involves nonoperative management. We report a case of successful nonsurgical treatment of a patient with extensive air involving the peritoneum, retroperitoneum, thorax, mediastinum, and subcutaneous tissues following an ERCP perforation.

## 1. Introduction

Endoscopic retrograde cholangiopancreatography (ERCP) has developed into an essential part of contemporary gastrointestinal practice since its introduction in 1968 [1]. In 2007, it was estimated that 500,000 ERCPs were performed annually in the United States [2]. Clinical indications for ERCP are diverse and include treatment of biliary tract disorders such as choledocholithiasis, strictures, and bile leaks; pancreatic disorders such as strictures, cancer, pseudocysts, or leaks; as well as ampullary disorders such as adenomas or sphincter of Oddi dysfunction [3]. Although ERCP is undoubtedly an invaluable component of interventional endoscopy, its use is limited by a complication rate of approximately 4–10% and mortality of 0.4% [2, 4–7]. Complications of ERCP, in order of frequency, include pancreatitis (1.3–5.4%), hemorrhage (0.76–3%), cholangitis (0.87–1%), and perforation (0.3–2.1%). The risk of complications is statistically increased in therapeutic as compared to diagnostic ERCP at rates of 1.38% and 5.4%, respectively, and, increasingly, ERCP is performed primarily for therapy. 

Perforation is a potentially serious complication of ERCP, leading in some cases to peritonitis, sepsis, and even death. As a result, most perforations after ERCP historically were treated with surgical repair. However, nonsurgical management has been shown to be increasingly successful in the management of most perforations, except for those that occur distant from the ampulla or bile ducts. We report a case of perforation as a complication of ERCP which resulted in large quantity of extraluminal air and was managed successfully without surgical intervention.

## 2. Case Report

A 73-year-old female was hospitalized for sudden onset of abdominal pain, jaundice, elevated aminotransferases, and hyperbilirubinemia. She had previous gastrointestinal surgeries with cholecystectomy and choledochoduodenostomy for congenital biliary obstruction at six months of age and gastroduodenostomy for bowel obstruction due to adhesions at five years. Computed tomography (CT) of the abdomen with contrast revealed dilated intrahepatic ducts and a 3.7 × 2.9 cm cystic structure adjacent to duodenum. On percutaneous cholangiography (PTC), a stricture was seen at the junction of the bile duct remnant and the cystic structure. A single percutaneous stent was placed for initial biliary decompression, and then two internal biliary stents were placed by combined PTC-ERCP. Repeated biliary brushings and biopsies of the stricture did not detect malignancy.

The ERCP of note was performed two months later. After extraction of the previously placed biliary stents, cholangiogram showed a persistent biliary anastomotic stricture. Balloon dilatation was performed and then a total of four stents, two 10 Fr. and two 8.5 Fr., were placed across the anastomosis to maintain patency. The patient remained stable throughout the procedure, with close monitoring by anesthesiology, and no complications were noted.

**Figure 1:**
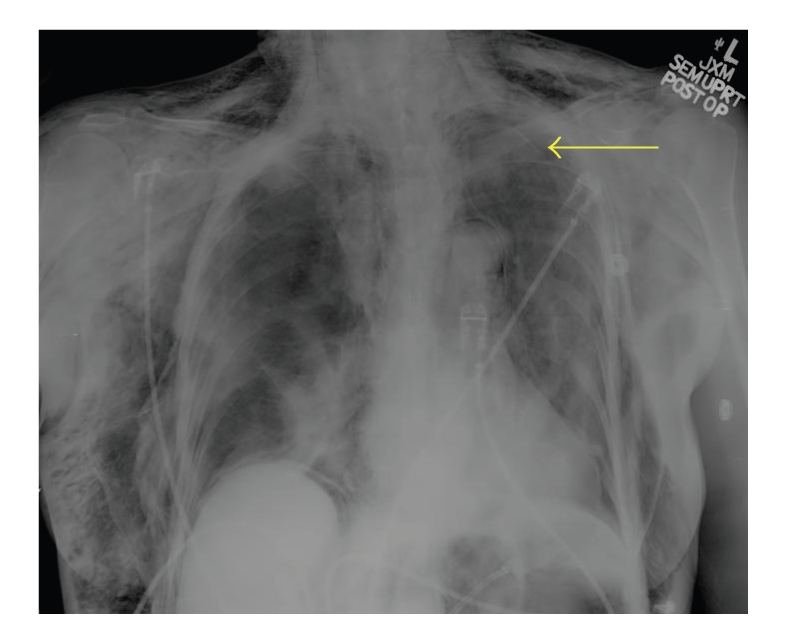
Portable chest X-ray showing subcutaneous emphysema, pneumomediastinum, and a left pneumothorax (arrow).

**Figure 2:**
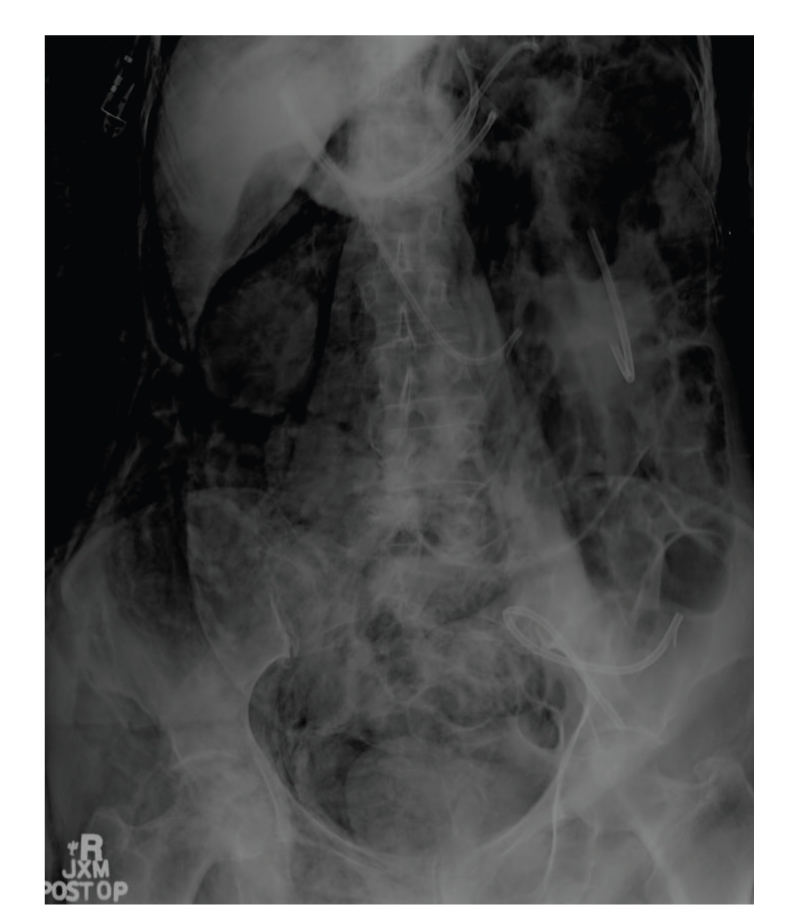
Portable abdominal X-ray showing retroperitoneal and intraperitoneal-free air.

Postoperatively, the patient developed dyspnea and right-sided, nonradiating chest pain. Despite normal oxygen saturation on room air, supplemental oxygen via non-rebreather face mask at 100% provided no relief of her symptoms. She denied any hematemesis, hemoptysis, cough, dysphagia, or abdominal pain. She was afebrile and mildly hypertensive, without tachycardia or tachypnea. Physical exam revealed subcutaneous emphysema of the neck, thorax, abdomen, and proximal upper and lower extremities. Her heart and lung sounds were notably decreased. She had mild abdominal distention, but no peritoneal signs. The remainder of her examination was normal. Laboratory studies, including arterial blood gas, complete blood count with differential, basic metabolic panel, and cardiac enzymes, were unrevealing. Her electrocardiogram showed normal sinus rhythm without any acute changes. Portable chest and abdominal X-rays revealed subcutaneous emphysema, pneumomediastinum, small left pneumothorax, and retroperitoneal and intraperitoneal free air (Figures 1 and 2).

**Figure 3:**
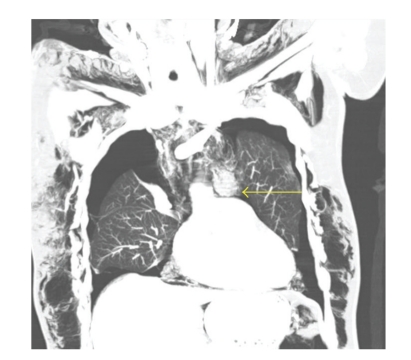
Coronal section of CT thorax showing subcutaneous emphysema, bilateral pneumothorax, and pneumomediastinum (arrow).

**Figure 4:**
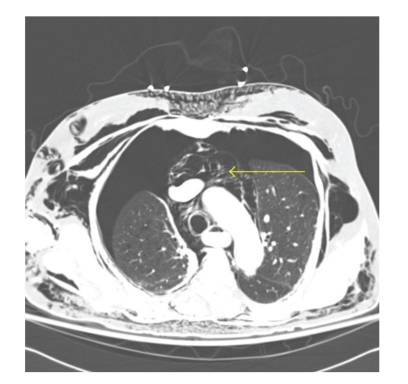
Transverse section of CT thorax showing subcutaneous emphysema, bilateral pneumothorax, and pneumomediastinum (arrow).

**Figure 5:**
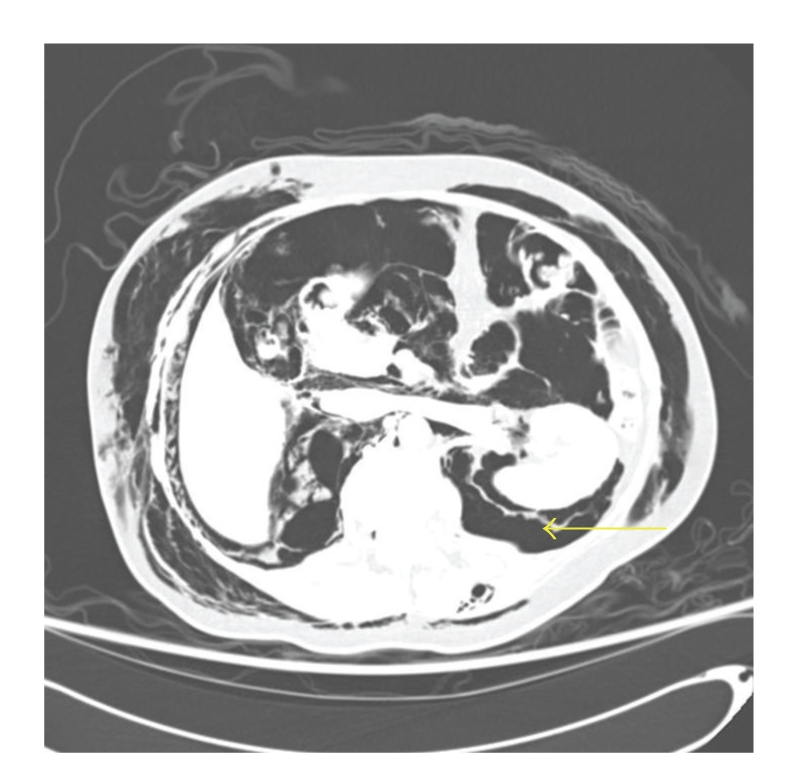
Transverse section of CT abdomen showing subcutaneous emphysema, pneumoperitoneum, and pneumoretroperitoneum (arrow).

**Figure 6:**
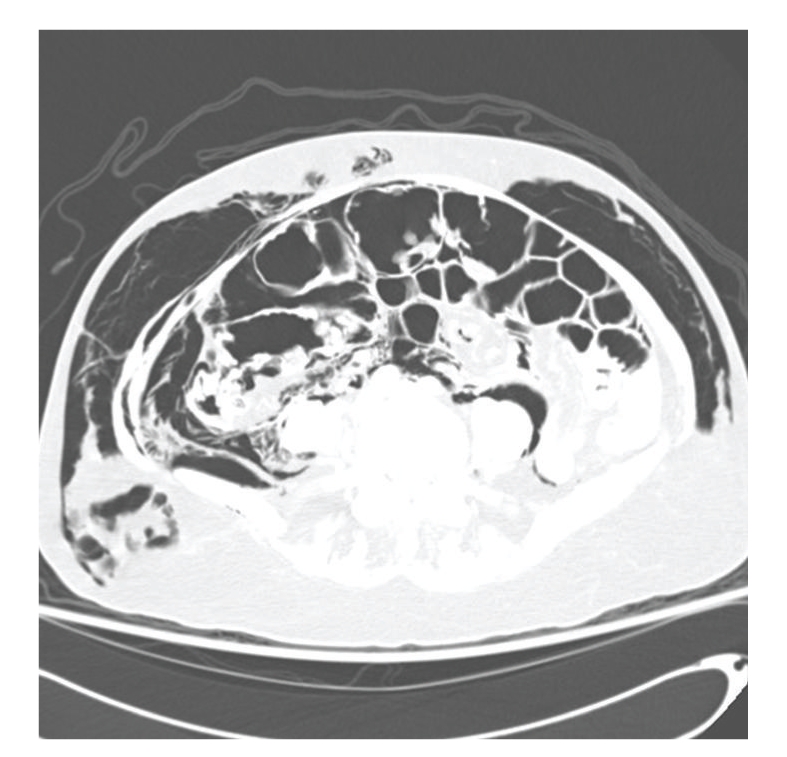
Transverse section of CT abdomen showing subcutaneous emphysema and pneumoperitoneum.

CT scan with intravenous and oral contrast of the thorax, abdomen and pelvis revealed diffuse subcutaneous emphysema (Figures 3–6), bilateral pneumothorax (Figures 3 and 4), pneumomediastinum (Figures 3 and 4), pneumoretroperitoneum (Figure 5), and pneumoperitoneum (Figures 5 and 6). The patient's solid organs were normal and there was no extravasation of oral contrast into the abdomen. 

The patient was admitted to the intensive care unit where she started on broad spectrum antibiotics and bowel rest. She underwent bilateral chest tube placement for decompression of the pneumothroaces. Her dyspnea and chest pain promptly resolved. Additional diagnostic studies, including esophagram, nasopharyngoscopy, and laryngoscopy, showed no gross site of perforation. Serial abdominal and radiographic examinations over the next three days showed partial resorption of peritoneal air. The patient remained hemodynamically stable throughout her treatment. Chest tubes were removed after 3 days, diet advanced without difficulty, and the patient was discharged uneventfully after one week. On follow-up visits, the patient was doing well with no residual symptoms. The biliary stents were removed by a final ERCP four months later. Cholangiogram at that time showed resolution of the anastomotic stricture with effective spontaneous biliary drainage.

## 3. Discussion

### 3.1. Epidemiology

Perforation is an uncommon complication of ERCP, with an incidence between 0.3% and 2.1% of procedures [2, 4–6, 8–16]. Therapeutic ERCP with sphincterotomy has a much higher rate of perforation than diagnostic ERCP (0.8–0.98% and 0.03–0.1%, resp.) [4, 8, 9]. Risk factors for perforation include older age, difficulty and length of the procedure, length of sphincterotomy, periampullary diverticulum of the duodenum, abnormal anatomy (Billroth II gastrectomy), dilated bile duct, biliary duct stricture, sphincter of Oddi dysfunction, and papillary stenosis [16–18]. In one study, the risk of perforation increased by 1.26 times for every 10 minutes over the mean time for procedure completion [12]. A papillotomy incision beyond the recommended 11 to 1 o'clock position also carries an increased risk [6]. There is controversy over whether precut papillotomy increases the risk of perforation as compared to sphincterotomy alone [17]. In one study, precut papillotomy was performed in 54% of patients with subsequent ERCP-related perforations [12]. 

Although the incidence of ERCP-related perforations is low, mortality has been reported in up to 20% of patients [2, 4, 9, 14, 16]. In cases where nonsurgical management failed, mortality increased to 50% [2, 10]. As a result of spillage of intestinal, biliary, and pancreatic contents into the abdomen, the most common cause of death is sepsis. A difference in outcomes after perforation exists between patients who were treated without surgery and those who underwent surgery. This included length of hospital stay, with a mean of 7 days in those medically managed versus 12–21 days in those surgically managed [8, 14], and mortality (4% versus 13% in nonsurgical versus surgical management of patients, resp.) [9, 14]. Patients who had a delay in diagnosis and operative therapy (≥12–24 hours after ERCP) had no difference in length of hospitalization and complications compared to those who initially had symptoms significant enough to require surgery, emphasizing the importance of early diagnosis and management.

### 3.2. Classification and Pathophysiology

There are two main classification systems for ERCP-related perforations. Howard et al. proposed a 3-group classification system based on the mechanism of ERCP-related perforation [11]. Group I in the Howard classification refers to guidewire perforations, group II periampullary, and group III duodenal perforations. Alternatively, Stapfer et al. classified perforations into 4 types based on severity and anatomical location [10]. The two systems are, for the most part, interchangeable. The Stapfer classification includes the following:

1. type I: lateral or medial duodenal wall perforations (Howard group III),
2. type II: peri-Vaterian injury (Howard group II),
3. type III: bile or pancreatic duct injury (comparable to Howard group I since majority of these perforations are caused by guidewire instrumentation),
4. type IV: presence of retroperitoneal air alone.

Stapfer type I perforations are due to the endoscope itself, tend to be large, and are usually intraperitoneal. Manipulation of the ampulla during sphincterotomy or other therapeutic measures causes type II perforations, which is the most common type of injury [13]. Guidewire (type III) perforations typically occur in the distal bile duct after wire or basket instrumentation near an obstruction. Type IV injuries generally are not gross perforations. Several studies have shown that retroperitoneal air seen on CT scan occurs in up to 29% of asymptomatic patients after an ERCP [19, 20]. These are caused by the use of compressed air to maintain patency of the duodenum during the procedure, regardless of the length of the procedure. 

Any perforation may present with retroperitoneal air that tracks to the thorax and subsequently into subcutaneous tissues, thus causing subcutaneous emphysema. It has been hypothesized that there are pores in the diaphragm, formed either congenitally or acquired, that allow air to move between the abdominal and thoracic cavity [21]. Others suggest that trauma to the duodenal wall by the endoscope allows insufflated air to enter the mucosa and track along the perineural and perivascular sheaths to enter the mediastinum [22]. In addition, the visceral space of the deep cervical fascia in the neck surrounds the trachea and esophagus and is contiguous with the diaphragmatic/esophageal hiatus, hilar vessel interstitium, and major airways of the thorax [23]. Therefore, this space allows the movement of air to flow between the retroperitoneum, mediastinum, and subcutaneous tissues of the neck. This permits subcutaneous emphysema to form around the upper cervical region, which then tracks down the endothoracic fascia of the chest wall to the transversalis fascia of the abdomen to cause diffuse subcutaneous emphysema, as was seen in this case report.

### 3.3. Diagnosis

A high clinical suspicion is essential for diagnosing ERCP-related perforations to allow for early diagnosis and subsequent optimal management and better prognosis. With the use of sedation, older age of patients, and chronic multiple comorbid medical issues, symptoms may initially be masked, making the diagnosis more difficult [14, 24]. Early diagnosis increases the chance that the patient will initially be treated nonsurgically, which results in shorter hospital stays and less complications [8, 18]. Perforation should be suspected in any patient with abdominal symptoms, chest pain, shortness of breath, or crepitus following an ERCP. Since pancreatitis is the most common ERCP complication and can present with similar symptoms, it must also be considered on the differential.

Diagnosis can occur during the ERCP if extravasation of air or contrast is seen outside the bile ducts and duodenum into the retroperitoneal space. In addition, abnormal guidewire position on fluoroscopy may also indicate perforation [11, 17]. In cases of type I perforations, the peritoneum or abdominal contents may be visualized during the procedure since the injury is usually large.

The clinical presentation of patients with perforation in the postprocedure period is usually nonspecific. One study performed a prospective analysis of patients with perforation after ERCP found that 100% of patients with perforation had abdominal or flank discomfort, 74% had elevated heart rate, 64% had mild to moderate abdominal tenderness, 47% had low-grade fever, 37% had hyperamylasemia (amylase >150 U/L), 32% had mild leukocytosis (WBC count 10,000–12,000/microliter), 18% had peritoneal signs, and 16% had subcutaneous emphysema [18]. Usually the degree of hyperamylasemia is not as elevated as expected in ERCP-related pancreatitis [6]. In a meta-analysis, a majority of patients present initially with mild epigastric tenderness that leads to fever and tachycardia, then peritonitis after several hours [17]. A retrospective study used a clinical score to compare patients that underwent operative versus nonoperative management of the perforation [8]. The clinical index was comprised of giving one point for each of the following: fever (≥38.5°C), tachycardia (heart rate ≥100 bpm), abdominal guarding on physical examination, and leukocytosis (WBC count ≥ 10,000). They found that 83% of patients medically managed had a score of 0 to 1, while 83% of patients that required surgery had a clinical index score of 3 to 4 (odds ratio for requiring surgery in patients with a score of 3 to 4 was 40). Although this clinical index has not been validated prospectively, it emphasizes the important clinical findings that help to guide therapy in patients with suspected perforation. A higher American Society of Anesthesia (ASA) classification also correlated with a higher likelihood of requiring surgical management in patients with ASA score ≥3 [9].

Because of its ease of administration, the first imaging study performed is usually an abdominal X-ray. This may demonstrate retroperitoneal air as streaking opacities in the right upper quadrant and outlining the kidney margins and along the psoas muscle [17, 23, 25]. Full expiratory upright abdominal X-rays can distinguish between intraperitoneal and retroperitoneal air, as the intraperitoneal air will decrease and the latter will increase with this maneuver [23]. Less common and specific findings on X-ray include obliteration of the psoas margin and segmental ileus in or near the duodenum. Abdominal CT scans usually confirm the diagnosis of perforation. It is believed that the air in the retroperitoneal and intraperitoneal space tracks along inferior vena cava to enter the mediastinal and pleural cavities, resulting in pneumomediastinum and pneumothorax, respectively. This can be seen on the CT scans of patients with perforations. Importantly, the amount of air seen on radiographs does not correlate with the severity of the disease but usually relates to the amount of manipulation performed after the perforation has occurred [6, 7]. Upper GI (UGI) series have also been performed on these patients and a diagnosis of perforation made with extravasation of contrast. However, this technique has a lower sensitivity than CT scans to detect microperforations and does not help to rule out other causes of similar symptoms, such as pancreatitis [18]. For this reason, abdominal CT without contrast is considered the radiographic imaging of choice to detect ERCP-related perforations in a patient that has abdominal pain or signs of systemic inflammatory response and peritonitis. Even with all of these radiographic studies, a source of the perforation may not be detected in up to 10% of cases [26]. 

### 3.4. Management

Treatment of ERCP-related perforations depends on the type of injury and the patient's clinical symptoms. All type I perforations, since they are usually large, are immediately repaired with general surgery [10, 11, 17, 27].The type of surgery depends on the extent of perforation and ranges from oversewing with omental patch to pyloric exclusion, gastrojejunostomy, tube duodenostomy, and extensive debridement. Type III perforations usually close spontaneously and may be conservatively managed or treated with placement of a biliary stent [11, 27]. Because type IV perforations demonstrate only retroperitoneal air and are not true perforations, their course is self limited and they too are generally managed without surgery. In a recent case report, a patient with Type IV perforation who had extensive air extravasation was managed without operative intervention [28].

The approach to management of type II perforations is less clear. Most tend to seal spontaneously by 2-3 days, but 10–43% of patients may require surgical repair [10, 15, 29]. Radiographic findings that require surgery include retroperitoneal or peritoneal fluid on CT, which suggests continued bile leak from site of perforation, and are associated with worse prognosis, large contrast extravasation on ERCP or UGI, and possibly the presence of massive subcutaneous emphysema. Clinical findings that require surgery include peritoneal signs and sepsis, which may be masked since most of these injuries are retroperitoneal. If none of these findings are present, then the patient may be closely monitored and managed with nothing by mouth, intravenous (IV) hydration, broad-spectrum IV antibiotics (antifungals may be added if the clinical course exceeds 3 days), and serial abdominal exams and radiographic imaging. In some cases, the patient also may benefit from diversion of bile and pancreatic secretions from the site of perforation using a biliary stent or nasobiliary tube, particularly when there are signs of biliary obstruction or cholangitis [10, 11, 18, 27]. Once the patient demonstrates improvement clinically, the diet may be initiated and slowly advanced. If a biliary stent is placed, then it can be removed electively.

Our patient serves as an excellent example of nonsurgical management of a Stapfer type II or III perforation. The likely cause of perforation was dilation and stenting at the anastomosis of common hepatic duct and duodenal diverticulum or choledocele remnant, which can best be understood as a biliary perforation at the surgical anastomosis. The initial presentation might readily have led to unnecessary surgical intervention with massive subcutaneous air, symptoms of chest pain and dyspnea, and the radiologic findings of marked retroperitoneal, intraperitoneal, mediastinal air and bilateral pneumothoraces. However, with chest tube placement, all symptoms resolved and the patient's clinical course was entirely uneventful. With close observation and patience, her hospital stay was shortened and she avoided the additional risks and morbidity of surgical intervention.

## 4. Conclusions

ERCP-related perforation is uncommon but has a high mortality rate, making it a feared complication. Because it usually presents with clinical findings similar to those of pancreatitis, a high clinical suspicion is needed to recognize perforation and initiate therapy promptly to achieve better outcomes. Stapfer type I perforations routinely require surgery and type IV perforations can be managed with observation alone. Treatment of Stapfer types II and III must be individualized based on the clinical and radiographic features of the patient. In this case of type II or III perforation, massive subcutaneous emphysema and extensive air throughout the abdomen and chest might have indicated surgery, but the outcome was very good with nonoperative management. As experience grows with the conservative management of perforation after ERCP, surgery may be required only for the most compelling indications of fluid extravasation, peritonitis, or sepsis.

## Figures and Tables

**Algorithm 1:**